# Aortic Root and Tricuspid Annulus Reconstruction for Transcatheter Valve Replacement–Associated Infective Endocarditis in an Octogenarian

**DOI:** 10.1016/j.jaccas.2026.107635

**Published:** 2026-03-28

**Authors:** Harod A. Silva, Yemmy Perez-Valverde, Edwin A. Masabel, Van Dyck Hector, Josias C. Ríos Ortega

**Affiliations:** Cardiovascular Surgery Department, National Cardiovascular Institute, EsSalud, Lima, Peru

**Keywords:** aortic root replacement, infective endocarditis, transcatheter aortic valve replacement, tricuspid annulus reconstruction

## Abstract

**Background:**

Prosthetic valve endocarditis after transcatheter aortic valve replacement (TAVR) is a rare and life-threatening complication, particularly in elderly patients.

**Case Summary:**

An 88-year-old man with a previous TAVR was readmitted 2 months after the procedure with fever (38.6 °C). Blood cultures were positive for methicillin-resistant *Staphylococcus epidermidis*. We performed extensive surgical debridement and reconstruction of the aortic root and tricuspid annulus, followed by a Bentall procedure and tricuspid valve replacement. The postoperative course was favorable, with good early recovery and normally functioning prostheses at follow-up.

**Discussion:**

This case illustrates that, even in octogenarian patients with prohibitive surgical risk and extensive multivalvular involvement, aggressive surgical management guided by a multidisciplinary heart team may provide favorable outcomes when medical therapy alone fails to control infection.

**Take-Home Messages:**

TAVR-associated prosthetic valve endocarditis may present with extensive perivalvular and multivalvular involvement. Surgical treatment, although rarely performed in this setting, can be lifesaving when guided by careful patient selection and multidisciplinary evaluation.

## Visual Summary

**Figure:**
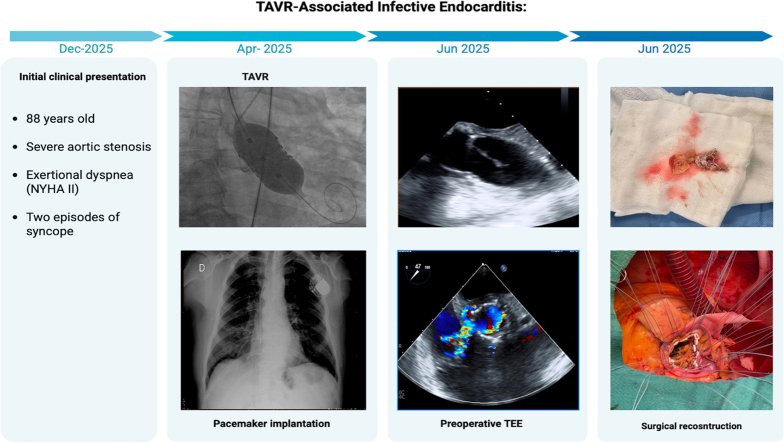

Transcatheter aortic valve replacement (TAVR) represents an alternative treatment for patients with symptomatic severe aortic stenosis deemed to be at prohibitive or high surgical risk, and the number of interventions has continued to grow each year.^1^ Prosthetic valve endocarditis (PVE) after TAVR is a rare but severe complication associated with poor clinical outcomes.^2^ Approximately 90% of these patients are managed conservatively with antibiotic therapy, which is associated with high in-hospital mortality and poor short-term prognosis.^3^ Only a few reports have described surgical treatment in very elderly patients with TAVR-PVE, particularly in cases involving multivalvular disease. This clinical scenario remains especially challenging given the high burden of comorbidities in this population.

**Take-Home Messages**

• TAVR-associated prosthetic valve endocarditis may present with extensive perivalvular and multivalvular involvement.
• Surgical treatment, although rarely performed in this setting, can be lifesaving when guided by careful patient selection and multidisciplinary evaluation.

We present a successful case of surgical treatment in an octogenarian diagnosed with infective endocarditis involving both the aortic root and tricuspid annulus after TAVR.

**Visual Summary:**
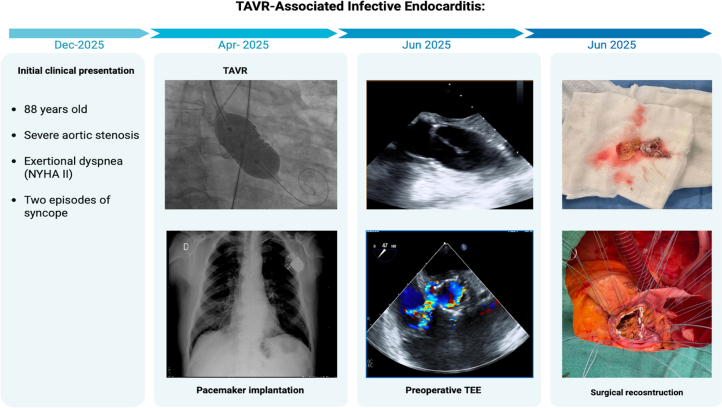
Timeline of Case Presentation TEE = transesophageal echocardiography; TAVR = transcatheter aortic valve replacement.

## History of Presentation

An 88-year-old man, previously diagnosed with severe aortic stenosis, underwent transfemoral TAVR with implantation of a 24.5-mm balloon-expandable valve (Myval, Meril Life Sciences). A prophylactic dose of cefazolin (2 g) was intravenously administered before the procedure.

During the postoperative period, the patient developed third-degree atrioventricular block requiring permanent pacemaker implantation. He was discharged 9 days after the procedure.

**Figure 1:**
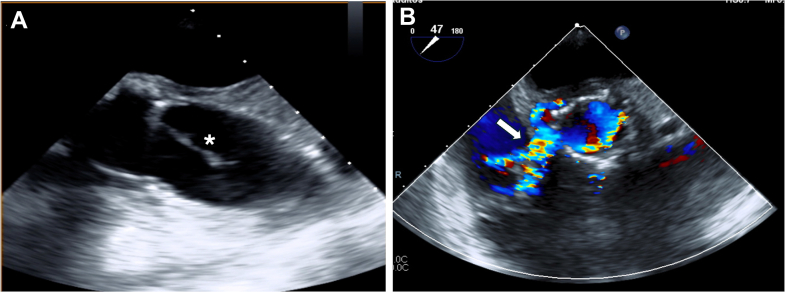
Preoperative Transesophageal Echocardiography (A) Transesophageal echocardiography showing vegetation attached to the aortic prosthesis (asterisk). (B) Color Doppler showing fistulous communication between the noncoronary sinus and the right atrium (arrow).

The patient was readmitted 2 months later with fever (38.6 °C). Blood cultures revealed methicillin-resistant *Staphylococcus epidermidis* bacteremia, and treatment with vancomycin was initiated for 14 days prior to any new surgical procedures; however, there was no significant reduction in inflammatory markers. Transesophageal echocardiography (TEE) revealed vegetations on the aortic prosthesis, the largest measuring 23 × 7.8 mm, as well as perivalvular thickening, with a hypoechoic area suggestive of abscess formation (Figure 1A). In addition, a fistula was identified between the noncoronary sinus and the right atrium, and a mobile vegetation was observed on the septal leaflet of the tricuspid valve (Figure 1B).

## Past Medical History

The patient had a history of hypertension. Six months before admission, he had experienced 2 episodes of syncope and had developed progressive exertional dyspnea (NYHA functional class II). He did not report any other relevant medical history.

## Differential Diagnosis

In the setting of persistent fever 2 months after TAVR and permanent pacemaker implantation, the differential diagnosis included TAVR-associated PVE with perivalvular extension (abscess/fistula) and cardiac implantable electronic device infection (pacemaker lead endocarditis) with secondary bacteremia.

## Management

The patient's estimated Society of Thoracic Surgeons score indicated a 20% mortality risk. A multidisciplinary heart team meeting was held, and surgical treatment was recommended.

**Figure 2:**
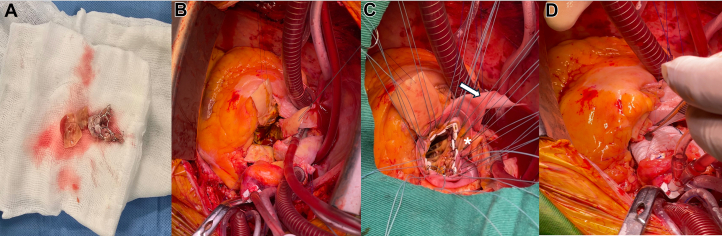
Intraoperative Findings (A) Transcatheter aortic prosthesis with attached vegetations. (B) Debridement of the aortic root and tricuspid septal leaflet. (C) Reconstruction of the aortic root (the asterisk indicates the neo-annulus) plus repair of the interatrial and interventricular septum (arrow) with a bovine pericardial patch. (D) Bentall procedure.

After median sternotomy and bicaval cannulation, cardiopulmonary bypass was established. An oblique aortotomy was performed, and cardioplegic solution was administered through the coronary ostia. The vegetation was carefully removed, and the transcatheter Myval prosthesis was explanted under direct vision, revealing complete destruction of the aortic annulus (Figure 2A). Radical debridement of all infected and necrotic tissue involving the aortic annulus and aortic root was then performed. Subsequently, a right atriotomy was carried out: The pacemaker leads and vegetation attached to the septal leaflet of the tricuspid valve were removed, the fistulous tract between the noncoronary sinus and the right atrium was opened, and the septal portion of the tricuspid annulus was resected (Figure 2B).

Surgical reconstruction of the aortic annulus was initiated using a bovine pericardial patch (Figure 2C), followed by a Bentall procedure (Figure 2D), with implantation of a 23-mm valved bioconduit. A separate patch was used to repair the tricuspid annulus and interatrial septum, and a 29-mm porcine bioprosthesis was implanted in the tricuspid position.

The patient was weaned from cardiopulmonary bypass uneventfully. Cardiopulmonary bypass time was 263 minutes; cross-clamp time was 226 minutes.

## Outcomes and Follow-Up

Postoperative TEE showed both prostheses with proper position and normal biventricular function. Intravenous antibiotic therapy was continued according to blood culture results for 42 days. A leadless pacemaker was successfully implanted.

Thirty days after surgery, follow-up TEE confirmed normally functioning prosthetic valves.

## Discussion

PVE after TAVR is a rare and life-threatening complication, with a reported annual incidence between 0.3% and 1.2%. In-hospital mortality ranges from 16% to 47%, and 1-year mortality from 27% to 75%, regardless of the management strategy.^2^ The most common causative microorganism in TAVR-PVE are enterococci (25%), *Staphylococcus aureus* (20%-25%) and coagulase-negative *Staphylococcus* (15%-20%).^2^^,^^4^

Surgical treatment is performed in only 2% to 14% of cases, mainly because of the patient's advanced age, high surgical risk, local complications (eg, abscess formation), and limited life expectancy.^5^ Although conservative antibiotic therapy can be an appropriate strategy in selected cases of TAVR-PVE without abscess or local complications, the presence of annular abscess and fistulization in our patient made medical therapy alone insufficient, thus justifying surgical intervention. In our case, despite a prohibitive Society of Thoracic Surgeons risk score (20%), surgery was performed given evidence of uncontrolled infection and perivalvular extension.

The main principles of success in these patients are extensive and complete debridement of all infected and compromised tissues. In our case, we performed radical resection and debridement of aortic root and tricuspid annulus, followed by reconstruction using 2 bovine pericardial patches and continuous monofilament sutures to avoid tension and reduce the risk of postoperative dehiscence.

There are only a few reports of successful outcomes after surgical treatment for TAVR-PVE in octogenarian patients with multivalvular involvement, particularly in cases with concomitant aortic root and tricuspid annular destruction. The present case demonstrates that surgical indications must be individualized and guided by multidisciplinary heart team evaluation, taking into account comorbidities and overall prognosis. When feasible, surgery can lead to favorable outcomes even in patients at prohibitive operative risk.

## Conclusions

Radical surgical reconstruction can be a viable rescue strategy in selected octogenarian patients with complex TAVR-associated infective endocarditis when medical therapy fails. This case supports an individualized, heart team–driven approach based on the anatomical extent of disease, hemodynamic impact, and infection control rather than chronological age alone.

## Funding Support and Author Disclosures

The authors have reported that they have no relationships relevant to the contents of this paper to disclose.
